# PET Testing Has Utility in the Prescription of Peritoneal Dialysis: PRO

**DOI:** 10.34067/KID.0000000000000434

**Published:** 2024-04-04

**Authors:** Martin J. Schreiber Jr

**Affiliations:** DaVita Kidney Care, Integrated Kidney Care, Denver, Colorado

**Keywords:** dialysis, ESKD, peritoneal dialysis, peritoneal membrane

## Introduction

The peritoneal equilibration test (PET) was first introduced into clinical practice by Twardowski in 1987^1^ and has traditionally been deemed a key contributor to effective peritoneal dialysis (PD) management. However, more recently, there has been a growing debate among nephrologists as to the clinical value of the PET in prescribing PD.^2^ A number of nephrologists possessing broad clinical experience in PD are able to empirically design the initial prescription and then adjust, as needed in a trial and error approach, without the benefit of a PET.

And yet, there is a significant percentage of seasoned nephrologists, as well as, new trainees completing fellowship and entering nephrology practices, especially in the United States, who have little experience in prescribing and maintaining patients on PD. This inexperience phenomena is also amplified in the setting of small PD programs (<20 pts.) and by the steady exodus of experienced PD nurses, most dramatic surrounding the coronavirus pandemic, who traditionally provided significant support and teaching, to less experienced clinicians. This lack of clinical experience, coupled to the “Advancing American Kidney Health” Executive Order^3^ which promotes home dialysis and transplantation, could negatively affect home dialysis growth. Therefore, the information gleaned from the PET would provide important pathophysiologic insights into prescribing PD, especially for inexperienced prescribers, increasingly important today with this historic focus on home dialysis.

## Understanding the Application of PET to Prescription Management

Data from a properly conducted PET provides pathophysiological insights, which can guide the design of the initial PD prescription, leads to a better understanding of which prescription changes can address inadequate solute removal or volume challenges, and provides identification of at-risk patients, that if proactively addressed could avoid unnecessary treatment complications. In general, data acquisition and interpretation of key tests is critical for managing complex patients such as experienced and patients with ESKD. As noted in a Canadian population-based study, patients with kidney disease have the highest mean number of comorbidities, the highest mean number of prescribed medications, the highest rate of death, and placement in long-term care facilities.^4^

From a pathophysiologic standpoint, the International Society of PD recommended a framework for amechanistic approach to peritoneal membrane dysfunction shifting terminology to focus on fast-peritoneal solute transfer rate (PSTR), low ultrafiltration (UF) capacity, and a diagnostic approach to intrinsic membrane dysfunction.^5^ Conceptually, these up to date recommendations provide important data which assists the user in clinical decision making. Changes in small solute transport and alterations in UF can occur over time, with >30% of patients experiencing an increase in peritoneal permeability to small solutes, accompanied by a decrease in UF capacity after 3–4 years on PD; the PSTR (4 hours D/P creatinine) increases in approximately one of every three patients by at least 0.1 after 1 year on PD treatment.

On average, approximately 40% of PD patients drop out of PD programs/yr., with movement to hemodialysis accounting for a significant percentage of modality transitions. Multiple factors can contribute to premature patient dropout in PD, and yet, as is true in several other areas of nephrology with unanswered diagnostic or therapeutic questions,^6^ there are currently no well-designed studies which specifically examine dropout rates comparing programs using PET versus no PET. However, by recognizing the potential that PM transport characteristics have in identifying a cohort of high-risk patients^7^ for PD loss (*i.e*., fast PSTR) this should lead to a higher intensity of follow-up, mitigating strategies and prescription modification.

## Evolving Concept of PD Prescription Management

A number of alternate PET methods have been reported, with each emphasizing a specific advantage over alternate versions (fast PET, mini-PET, modified PET).^8^ Regardless, the standardization of a nephrology practice's/home program PET protocol should include the use of a 3.86% glucose/4.25% dextrose PET instead of a 2.27% PET whenever possible, use of creatinine as the index solute, measuring sodium sieving/sodium dip at 1 hour, and the UF capacity (observed UF of >400 ml at 4 hours). Several studies demonstrated similar small solute transport comparing the use of 2.5% glucose dialysate, with the 4.25% PET, while providing the additional benefit for assessing UF capacity.^9^ While the classic PET used a 2-L dwell volume, there is support for adjusting the test dialysate volume to the volume prescribed as per the individual patient's body size.^10^

**Figure 1:**
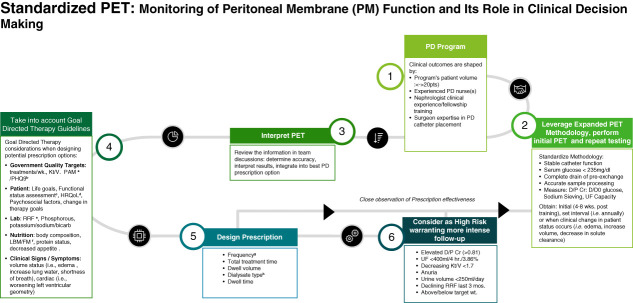
**PET monitoring plays a key role in the initial prescription design and at future time points (regular or on the basis of change in clinical status).** Proactively identify changes in PM function that predict or correlate with observed clinical findings. Goal-directed therapy, shared decision-making discussions, and increased attention to high-risk patients are important management strategies for improving quality life survival on PD. ^a^patient activation measure (PAM), ^b^patient health questionnaire (PHQ9), ^c^short physical performance battery (SPPB), FRIED frailty index (FP), physical activity scale for elderly (PASE), ^d^health related quality of life (HRQoL), ^e^residual renal function (RRF), ^f^lean body mass (LBM)/fat mass (FM), ^g^4–8 weeks, ^h^dextrose-based, icodextrin-based. PD, peritoneal dialysis; PET, peritoneal equilibration test.

Variations in patient results can be significantly reduced with greater standardization of data acquisition (Figure 1); this goal requires specific attention to catheter function, avoiding significant intra-abdominal residual dialysate in the exchange before conducting the PET exchange, complete PET drain, correct sampling of the dialysate and processing, and systemic serum glucose control in diabetic patients. Proactively obtaining the PET at specific intervals (q1−2 years) may be the best option in inexperienced programs, while other programs with larger patient populations, legacy PD nurses, and greater clinical experience may favor repeating the PET when issues arise, warranting validation of the clinical impression.

## Technology Innovations and PET

PD programs may benefit from user-friendly mathematical computer programs, such as Adequest, Pack-PD, and Personal Dialysis Capacity that assist in PD prescription design, based on an individual patient's transport PET data, residual renal function, and individualized requirements, *i.e*., total time on treatment, dwell volume tolerance. Both prescription modeling programs can be helpful to experienced and inexperienced programs for designing initial prescriptions, while aligning a patient's daily activities with the best prescription option. Going forward, artificial intelligence programs will play an increasing role in dialysis care. Machine learning, deep learning,^11^ and devising neural pathways will provide a roadmap for mimicking how nephrologists think, aiding in PD prescription design regardless of experience. Future innovations will have the potential to assimilate and analyze PD data across the entire spectrum of the health record, identify patients who warrant a PD prescription redesign, generate patient-sensitive PD prescription options, while leveraging predictive analytics to derive a virtual PET on the basis of accumulated dialysis treatment results and laboratory data, thus decreasing nurse time and patient burden.

Performing the PET is a key component to active PD patient monitoring, especially in PD programs with limited clinical experience. Future innovative approaches to digitally assist in big patient data integration for making individual patient clinical care decisions will play an increasing role in dialysis care. In closing, the true value of a test, like the PET, often lies not just in what it measures easily, but in what it reveals and how it adds to physician knowledge despite the challenges of measurement.
